# Instrumental variable-based high-dimensional mediation analysis with unmeasured confounders for survival data in the observational epigenetic study

**DOI:** 10.3389/fgene.2023.1092489

**Published:** 2023-02-02

**Authors:** Fangyao Chen, Weiwei Hu, Jiaxin Cai, Shiyu Chen, Aima Si, Yuxiang Zhang, Wei Liu

**Affiliations:** ^1^ Department of Epidemiology and Biostatistics, School of Public Health, Xi’an Jiaotong University Health Science Center, Xi’an, Shaanxi, China; ^2^ Department of Radiology, First Affiliated Hospital of Xi’an Jiaotong University, Xi’an, Shaanxi, China; ^3^ Department of Cell Biology and Genetics, School of Basic Medical Science, Xi’an Jiaotong University Health Science Center, Xi’an, Shaanxi, China

**Keywords:** high-dimensional mediation analysis, instrumental variable, unmeasured confounder, survival data, epigenetic study

## Abstract

**Background:** High dimensional mediation analysis is frequently conducted to explore the role of epigenetic modifiers between exposure and health outcome. However, the issue of high dimensional mediation analysis with unmeasured confounders for survival analysis in observational study has not been well solved.

**Methods:** In this study, we proposed an instrumental variable based approach for high dimensional mediation analysis with unmeasured confounders in survival analysis for epigenetic study. We used the Sobel‘s test, the Joint test, and the Bootstrap method to test the mediation effect. A comprehensive simulation study was conducted to decide the best test strategy. An empirical study based on DNA methylation data of lung cancer patients was conducted to illustrate the performance of the proposed method.

**Results:** Simulation study suggested that the proposed method performed well in the identifying mediating factors. The estimation of the mediation effect by the proposed approach is also reliable with less bias compared with the classical approach. In the empirical study, we identified two DNA methylation signatures including cg21926276 and cg26387355 with a mediation effect of 0.226 (95%CI: 0.108-0.344) and 0.158 (95%CI: 0.065-0.251) between smoking and lung cancer using the proposed approach.

**Conclusion:** The proposed method obtained good performance in simulation and empirical studies, it could be an effective statistical tool for high dimensional mediation analysis.

## 1 Introduction

Mediation analysis is widely used in exploring the internal mechanism of exposure on outcomes, especially in the epigenetic study (Vo et al., 2022). This methodology of mediation analysis was proposed to describe the relationship between exposure, mediating variables, and outcomes (Rijnhart et al., 2021). For clinical, epidemiological, or genomic studies, within the framework of the regression models, the effect of exposure on outcomes is decomposed into direct and indirect effects in mediation analysis (Lee et al., 2021).

Epigenetic modification refers to changes in gene expression or protein expression that do not involve changes in the DNA sequence. Epigenetic modifiers mainly include DNA methylation, histone covalent modification, chromatin remodeling, gene silencing, RNA editing, and other regulatory mechanisms. It plays an important role in the occurrence, development, and prognosis of cancer (Herceg and Vaissière, 2011). Epigenetic modifiers, such as DNA methylation, are often affected by environmental factors and are one of the important factors affecting the survival outcome of cancer patients. Considering the high-dimensional feature of epigenetic modifiers, such as DNA methylation, their roles between environment exposure and cancer survival were usually analyzed using high-dimensional mediation analysis. When the methodology of mediation analysis was first proposed, it was assumed that there were no confounding factors (Baron and Kenny, 1986); however, in observational studies focusing on the role of epigenetic modifiers, this assumption is hard to hold (Stuart et al., 2021).

Recently, several methodologies have been proposed for the analysis of high-dimensional mediation analysis (Dai et al., 2020; Zhang et al., 2021a; Yang et al., 2021; Perera et al., 2022; Wang et al., 2022; Zhao and Li, 2022; Zhao and Luo, 2022). Zhang et al. (2016) raised the issue of estimating the high-dimensional mediating effect in survival analysis (Zhang et al., 2016). Gao et al. (2019), Luo et al. (2020), and Zhang et al. (2021a) all proposed high-dimensional mediating analysis approaches based on penalty methods, and Cui et al. (2021) proposed a high-dimensional mediation analysis approach for survival data based on the addictive hazard model (Gao et al., 2019; Luo et al., 2020; Zhang et al., 2021b; Cui et al., 2021). These approaches have provided useful statistical tools for practical analysis; however, the issue of confounders remained (Stuart et al., 2021).

In general, the control of confounders in the observational study mainly adopts the frame of causal inference, such as using the propensity score (PS) method (VanderWeele, 2006). The development of the confounder adjustment methodology has greatly enriched the application of mediating effect analysis (Coffman, 2011; Valeri and VanderWeele, 2013). Yu et al. (2021) expanded Luo et al.’s (2020) approach (Luo et al., 2020) with the PS adjustment to control potential confounders (Yu et al., 2021). Liu et al. (2022) proposed a powerful divide-aggregate composite-null test (DACT) for causal mediation effects (Liu et al., 2022). Tian et al. (2022) proposed the CoxMKF approach to test high-dimensional mediating effects in survival data with confounders (Tian, et al., 2022). These published methods have provided useful statistical tools making it possible to estimate indirect effects in high-dimensional data survival controlling potential confounders.

The PS is the conditional probability of the individual in a specific exposure/treatment group estimated based on the level of the known confounding factors and is currently one of the most commonly used methods in the controlling of confounders (VanderWeele, 2006; Heinze and Jüni, 2011). The conduction of the PS method requires that all (at least the main) confounders are known and measured; however, it is not always true in practice, especially in observational studies (Armstrong, 2012). With the existence of unknown confounders, the efficacy of the PS approach would be seriously affected (VanderWeele, 2006; Heinze and Jüni, 2011; Armstrong, 2012). Therefore, the PS method will not always be able to guarantee a reliable estimation and inference when there are unmeasured confounders.

Instrumental variable (IV) analysis is commonly used to control bias caused by potential unknown confounders (Chen and Briesacher, 2011). The IV approach decomposes treatment/exposure into a part related to confounding factors and an irrelevant part to eliminate the influence caused by confounders (Chen and Briesacher, 2011). By isolating and using the part with no association with confounders, it is possible to estimate the association between the key explanatory variable and the outcome with the influence of potential confounders could be controlled using regression models (Chen and Briesacher, 2011). One of the many advantages of the IV approach is that it does not require the information of the confounders (Chen and Briesacher, 2011). It works as an effective alternative when the PS method does not work (Chen and Briesacher, 2011; Armstrong, 2012). The widely applied Mendelian randomization approach is also one of the most typical uses of the IV method which specifically refers to the use of genetic variation as IV to infer a causal relationship (Didelez and Sheehan, 2007). Tchetgen Tchetgen et al. (2015) implemented the IV method in time-to-event data analysis with the classic Cox regression model (Tchetgen Tchetgen et al., 2015). Li et al. (2014) applied the IV approach in the estimation of the additional hazard model (Li et al., 2014). Dippel et al. (2019) expanded the IV method into the analysis of the mediation effect with one mediator and one IV (Dippel et al., 2019). However, IV-based methods for high-dimensional mediation detection controlling potential unmeasured confounders, especially for the time-to-event outcome, have not yet been proposed.

In this study, we aim to propose an IV-based mediation analysis and an indirect effect estimation approach in high-dimensional mediation analysis for Cox regression models with unmeasured confounders. The rest of the paper is organized as follows. In the next section, we first briefly introduce the key idea, basic notation, definitions, assumptions, the IV approach, and propose the method. Then we conducted the simulation study to illustrate the statistical performance of the proposed method. We also compared the statistical performance of the proposed method, the PS method, and classical approach in estimation of indirect effects with existence of unmeasured confounders through the simulation study. Additionally, considering the high-dimensional nature of the data, the identification of IVs is also important. Therefore, we also compared different variable selection approaches in the screening of potential IVs in the simulation study. Then, a real data analysis was also conducted to show the application of the proposed method.

## 2 Statistical method

### 2.1 Definitions of models

**FIGURE 1:**
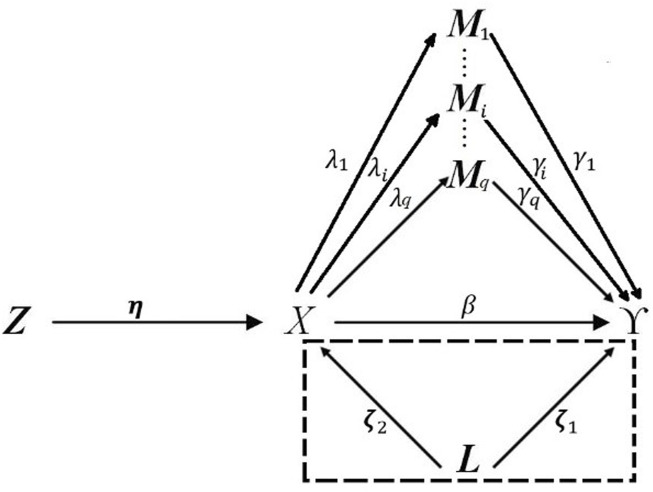
DAG describing high-dimensional mediation with IVs, and confounders affecting the relation between exposure, mediator, and outcome. The dotted box indicated that the confounders ***L*** may not be able to be measured in observational studies.

Let *X* and *Z*=(*Z*_1_, *Z*_2_,…, *Z*_k_) be the exposure variable and vector of IVs, respectively. The IVs may be continuous or binary variables, and *X* is a binary variable. The outcome variable Υ is time-to-event. Let *M*=(*M*_1_, *M*_2_,…, *M*_i_,…, *M*_q_) be the vector of normally distributed mediators with dimension q. Define n be the sample size, and *q* > *n*. Let *L*=(*L*_1_, *L*_2_,…, *L*_j_) be confounders that influence the relation between exposure X and outcome Υ . With a directed acyclic graph (DAG), we expand Dippel et al. (2020) mediation model (Dippel et al., 2020) to a high-dimensional situation with unmeasured confounders. The relationships between variables could be illustrated in Figure 1.

The aforementioned relations presented in Figure 1 can be expressed with the classic Cox regression model with mediators, confounders, and IVs asfollows:

$$h\left(t\right)=h_{0}\left(t\right)\exp\left(a+\beta X+\gamma^{T}M+\zeta_{1}^{T}L+\varepsilon_{1}\right)$$ (1)

$$M_{i}=c+\lambda_{i}X+\varepsilon_{2}\;\left(i=1,\ldots i,\ldots ,q\right)$$ (2)

$$logit\left(P\left(X=1\right)\right)=d+\eta^{T}Z+\zeta_{2}^{T}L+\varepsilon_{3}$$ (3)

where $\varepsilon_{\cdot }$ is the error term and $\varepsilon_{\cdot }∼N\left(0,\sigma^{2}\right)$ . β is the coefficient relating exposure *X* and outcome Υ . $\gamma =\left(\gamma_{1},\gamma_{2},\ldots ,\gamma_{q}\right)$ is the q-dimension coefficient vector relating the mediators ***M*** and outcome Υ . $\zeta_{1}=\left(\zeta_{11},\zeta_{12},\ldots ,\zeta_{1j}\right)$ is the j-dimension coefficient vector relating confounders ***L*** and outcome Υ . $\zeta_{2}=\left(\zeta_{21},\zeta_{22},\ldots ,\zeta_{2j}\right)$ is the j-dimension coefficient vector relating confounders ***L*** and exposure *X*. $\eta =\left(\eta_{1},\eta_{1},\ldots ,\eta_{k}\right)$ is the k-dimension coefficient vector relating IVs ***Z*** and exposure *X*. $\lambda =\left(\lambda_{1},\ldots ,\lambda_{i},\ldots ,\lambda_{q}\right)$ is the coefficient relating exposure *X* to *i*th mediator *M*_*i*_;_*.*_ *a*, *c*, and *d* are intercepts.

The parameters in Eqs. 1, 3 were estimated through the maximum likelihood estimation (MLE) approach while parameters in Eq. 2 were estimated through the ordinary least square (OLS) method.

### 2.2 Instrumental variable

The IV is used to help remove the influence of potential confounders, especially those unmeasured ones (Chen and Briesacher, 2011). Desirable IV is closely associated with exposure *X* and there is no direct relationship between IV and outcome variables (Chen and Briesacher, 2011). The outcome variables can only be affected by IV through exposure (Chen and Briesacher, 2011).

In general, IV analysis for linear models is estimated with the two-stage least square (2SLS) method. In the first stage, with the notifications in Eqs. 1–3, we use the IV to divide the exposure *X* into two parts as *X* = *D* + *V*, in which $D=d+\eta^{T}Z$ . Since IV is not associated with confounders, *D* is not affected by confounders either. For *V*, it is the part that cannot be explained by IV and is associated with confounders, which could be regarded as residuals ( $\varepsilon_{3}$ ). Then, the exposure *X* can be expressed as in Eq. 3. In the second stage, in non-mediation analysis, we could use Eq. 3 to replace the exposure in Eq. 1 and obtain the following equation:

$$h\left(t\right)=h_{0}\left(t\right)\exp\left\{a_{4}+\beta \left(\eta^{T}Z\right)+\gamma^{T}M+\zeta^{T}L+\varepsilon_{4}\right\}$$ (4)

As shown in Eq. 4, ***Z*** is not affected by confounders ***L***, and the IV approach allows the existence of unknown or unmeasured confounders by removing the association between potential measured or unmeasured confounders and the exposure.

### 2.3 Assumptions

To ensure the identification of the mediating effects, there are several assumptions that need to be hold for the methodology proposed in this study (VanderWeele, 2011; Huang and Yang, 2017; Yu et al., 2021; Tian et al., 2022).

A1. There are no confounders between the IVs and exposure.

A2. The mediators are independent of each other.

A3. There are no confounders between the mediators and the outcome.

A4. The IVs are not associated with any mediators.

### 2.4 Variable selection based on penalized approaches

Considering the presence of high-dimensional covariates, we need first to separate potential IVs from all available covariates. Several penalized approaches have been taken into consideration in the beginning, including the least absolute shrinkage selection operators (LASSO) (Tibshirani, 1996), the adaptive LASSO (ALASSO) (Huang et al., 2008), the elastic net (EN) (Zou and Hastie, 2005), and the MCP approach (Zhang, 2010), while the MCP approach yielded the best performance (details shown in the Simulation Study section).

### 2.5 Significance test for the mediation effect

We can identify the true mediator $M_{i}$ between the exposure and outcome from the potential mediator set M when the path-specific indirect effect is significant. Here, we used three methods to test whether the mediation effects between exposure X and outcome Υ are significant, including the joint significance test (MacKinnon et al., 2002), the Sobel’s test (Sobel, 1982), and the bootstrap test (Efron and Tibshirani, 1994).

The joint test is based on path-specific (i.e., X→M ; M→Υ ) indirect effect *p*-values. The *p*-value for the joint significance test is defined as follows:

$$P_{raw,i}=\max\left(P_{raw,\lambda_{i}},P_{raw,\gamma_{i}}\right)$$ (5)

where $P_{raw,\lambda_{i}}$ is the *p*-value for testing $H_{0}:\lambda_{i}=0$ of pathway X→M , and $P_{raw,\gamma_{i}}$ is the *p*-value for testing $H_{0}:\gamma_{i}=0$ of pathway M→Υ . In addition, we use the Bonferroni method to get an adjusted *p*-value for multiple comparisons as follows:

$$P_{adj,i}=\min\left(P_{raw,i}∙q,1\right)$$ (6)

where q is the number of potential mediators in set M.

The Sobel test focuses on the null hypothesis $H_{0}:\lambda_{i}\gamma_{i}=0$ of no indirect effect, that is, we tested whether the coefficient product of the pathway X→M and M→Υ is equal to zero or not. The *p*-value of the Sobel’s test is defined as follows:

$$P_{raw,i}=2\left\{1-\phi \left(\frac{\left|\hat{\lambda }\hat{\gamma }\right|}{{\hat{s}}_{\hat{\lambda }\hat{\gamma }}}\right)\right\}$$ (7)

where ${\hat{s}}_{\hat{\lambda }\hat{\gamma }}=\sqrt{{\hat{\lambda }}^{2}{S_{\gamma }}^{2}+\gamma^{2}{S_{\lambda }}^{2}}$ is the estimate of Sobel’s standard error, $\phi \left(∙\right)$ is the standard normal cumulative distribution function, $\hat{\lambda }$ and $\hat{\gamma }$ are the effect estimates of pathway X→M and M→Υ , respectively. Similarly, we can get the revised *p*-value *via* the Bonferroni approach as mentioned previously.

The bootstrap test obtains the asymmetric indirect effect $\left(1-\alpha \right)\%$ level confidence interval using the resampling method. Then we can reject the null hypothesis $\left(H_{0}:\lambda_{i}\gamma_{i}=0\right)$ when the confidence interval does not contain zero and conclude $M_{i}$ is the mediator between exposure X and outcome Υ . Here, we calculate the percentile bootstrap confidence interval. Given the original data with sample size n, the percentile bootstrap method is described as follows (Efron and Tibshirani, 1994):

Step 1: We obtained the bootstrap sample with sample size n by sampling with replacement from the original sample.

Step 2: We calculated the estimate of indirect effect $\lambda_{i}\gamma_{i}$ by using the bootstrap sample obtained previously.

Step 3: We repeated steps 1 and 2 for *B* times (usually *B* =1,000), then get B estimates of $\lambda_{i}\gamma_{i}$ .

Step 4: We constructed the confidence interval of the *B* estimates of $\lambda_{i}\gamma_{i}$ with a confidence level of 1−α using the percentile method.

Step 5: Then, we concluded the indirect effect $\lambda_{i}\gamma_{i}$ is not significant at α significance level if the confidence interval contains zero; otherwise, the $M_{i}$ is the mediator between exposure *X* and outcome Υ .

### 2.6 Proposed method

Considering that in observational studies, there is no guarantee that all confounders between the exposure and outcome can be measured, we propose the following high-dimensional mediation analysis method based on IV.

For the conduction of IV analysis, we need to select those variables associated with the exposure but not associated with others as candidate IVs. Then, we used the 2SLS method to conduct the regression-based IV analysis and estimated the effects of exposure on the outcome. Since the number of potential covariates (including IVs and mediators) is far more than that of the sample size, we need to reduce the dimensionality of the mediators to meet the requirements and capacity of the classic Cox and logistic regression models.

To ensure the efficacy of variable selection, we followed Luo et al. (2020) and Yu et al. (2021) and used the sure independence selection (SIS) (Fan and Lv, 2008) method to conduct a preliminary selection. Then we applied the MCP-based Cox regression model to select potential mediators followed by Luo et al. (2020) and Yu et al. (2021). For the selection of IVs, we compared four commonly used variable selection approaches in the simulation study (Table 1) and decided to use the MCP-based logistic regression.

**TABLE 1:** Performance of the four penalized approaches in the selection of IVs.

		LASSO		ALASSO		EN		MCP	
Censoring	20%	FDR	PSR	FDR	PSR	FDR	PSR	FDR	PSR
	200	0.007	0.820	0.006	0.796	0.031	0.905	0.005	0.892
Sample size	500	0.007	0.999	0.005	0.806	0.032	0.911	0.004	1.000
	800	0.009	1.000	0.005	0.823	0.034	0.961	0.004	1.000
Censoring	40%	FDR	PSR	FDR	PSR	FDR	PSR	FDR	PSR
	200	0.004	0.881	0.005	0.882	0.027	0.876	0.002	0.926
Sample size	500	0.005	0.984	0.008	0.911	0.036	0.913	0.003	1.000
	800	0.005	0.999	0.008	1.000	0.036	1.000	0.002	1.000
Censoring	60%	FDR	PSR	FDR	PSR	FDR	PSR	FDR	PSR
	200	0.004	0.989	0.008	0.881	0.026	0.889	0.003	0.855
Sample size	500	0.006	0.976	0.006	0.872	0.031	0.909	0.002	0.999
	800	0.009	1.000	0.009	0.889	0.037	0.999	0.002	1.000

Then, we estimate the indirect effects and corresponding standard errors between exposure to mediators, exposure to the outcome, and mediators to the outcome. At last, we test the mediation effect through the hypothesis test. For the hypothesis test of the mediation effect, we considered three commonly used approaches including the Sobel’s test, the joint test, and the bootstrap test. Also, considering the possible multi-comparison issue caused by the existence of multi-mediators, we used the Bonferroni approach to adjust the *p*-values.

The proposed approach can be summarized as follows:

Step 1: For all covariates, we use the SIS approach to preliminarily select variables associated with the exposure *X* and the outcome Υ . The selected variables are contained in subsets *I*_0_ (associated with exposure) and *M*_0_ (associated with the outcome). Both subsets are with a size of *t* = 2*n*/log(*n*), in which n is the sample size.

Step 2: With subset *I*_0_, we implement MCP-based logistic regression with exposure *X* being the dependent variable to select potential IVs. The selected variables are contained in set *I*_1_.

Step 3: With subset *M*_0_, we implement MCP-based Cox regression with the outcome ( Υ ) being the dependent variable to select potential mediators. The selected variables are contained in set *M*_1_.

Step 4: Variables in *I*_1_ but not in *M*_1_ were regarded as candidate IVs and contained in set *I*_2_. All variables in *M*_1_ were candidate mediators.

Step 5: We conduct a 2SLS-based IV analysis with exposure *X*, the outcome, and candidate IVs to estimate η between IVs and exposure *X*, γ between mediators and the outcome, and *β* between exposure and the outcome.

Step 6: With the estimated effects, we conduct the mediation analysis. The test of mediator and indirect effects is based on the hypothesis test methodologies including the joint test, the Sobel’s test, and the bootstrap method.

Step 7: *P*-values obtained were then adjusted through the Bonferroni approach. The adjusted *p*-value < 0.05 is considered statistically significant.

### 2.7 Evaluation of the performance of the proposed approach

To evaluate the statistical property of the proposed method, a comprehensive simulation study and an empirical study were conducted. The data used in the empirical study were obtained from The Cancer Genome Atlas (TCGA) database (https://portal.gdc.cancer.gov/ ).

## 3 Simulation study

### 3.1 Simulation design

To evaluate the statistical performance of the proposed method, we conducted a comprehensive simulation study. The implementation of the proposed method and simulation study was based on R-programming language (version 4.0.5, The R Foundation, Vienna, Austria) and the RStudio software (version 1.1.383, RStudio Inc., Boston, MA, United States). The main R packages used in the current study include “*survival*,” “*ncvreg*,” “*ggm*,” “*ivtool*,” “*glmnet*,” and “*boot*.” The choice of simulation parameters was based on published methodology studies and application studies focusing on the mediating role of epigenetic factors (Luo et al., 2020; Yu et al., 2021; Tian et al., 2022).

***Z*** =(*Z*_1_, *Z*_2_) are IVs, *Z*_1_ and *Z*_2_ were generated from a multi-normal distribution with $\mu_{i}=0\;\left(i=1,2\right)$ ; $\sigma_{1}=0.9$ ; $\sigma_{2}=1.1$ , and *cov*(*Z*_1_, *Z*_2_) = 0. For the (unmeasured) confounders ***L*** =(*L*_1_, *L*_2_, *L*_3_, *L*_4_), *L*_1_ and *L*_2_ were generated from a multi-normal distribution with $\mu_{i}=0\;\left(i=1,2\right)$ ; $\sigma_{1}=0.8$ ; $\sigma_{2}=1.2$ , and *cov*(*L*_1_, *L*_2_) = 0. *L*_3_ and *L*_*4*_ were generated from the Bernoulli distribution with parameters set as *p*_1_ = 0.4 and *p*_2_ = 0.6. The exposure *X* is generated based on the IVs and (unmeasured) confounders as defined in Eq. 3.

Then, the generation of the outcome variable was based on the method proposed by Wan (2016). The censoring rate was defined as 20%, 40%, and 60%, respectively. The coefficients vectors were defined as $\zeta_{1}=\left(0.4,0.5,0.6,0.7\right)$ , $\zeta_{2}=\left(0.4,0.5,0.6,0.7\right)$ , and *β* = 1.5. To evaluate the influence of the sample size, we chose three sample sizes of 200, 500, and 800. In the simulation study, we designed three scenarios.

#### 3.1.1 Scenario 1

We set the number of potential covariates (including the confounders, IVs, and exposures) equal to 1,000. Also, we denote $\gamma =\left(1.2,0.8,1.5,0,0,\ldots ,0\right)$ , $\lambda =\left(0.8,1.2,0,1.5,0,\ldots ,0\right)$ . Notably, $\lambda_{i}\gamma_{i}\neq 0$ indicated Mi is a significant mediator which means that there were two true mediators. The confounders were then removed to simulate the existence of unmeasured confounders.

#### 3.1.2 Scenario 2

We set the number of potential covariates equal to 3,000. Also, we denote $\gamma =\left(1.2,0.8,-1.2,1.2,1.5,0,0,\ldots ,0\right)$ , $\lambda =\left(0.8,1.2,0.8,-0.8,0,1.5,0,\ldots ,0\right)$ . Notably, $\lambda_{i}\gamma_{i}\neq 0$ indicated Mi is a significant mediator which means that there were four true mediators. The confounders were then removed to simulate the existence of unmeasured confounders.

An additional simulation study was also conducted to compare the statistical performance of the proposed method and other published approaches under the assumption that all confounders were measured. The results are presented in the supplementary file (Supplementary Table S1), the simulation parameters were the same as in Scenario 1 but the confounders were not removed. As shown in the supplementary file, the proposed method, the PS-based approach, and the CoxMKF method all achieved good performance.

### 3.2 Evaluation of the performance

The performance of the variable selection process was evaluated with the false discovery rate (FDR) and positive select rate (PSR) (Benjamini and Hochberg, 1995) which is defined as follows:

$$FDR=\left\{\begin{array}{c} \frac{FP}{FP+TP},FP+TP>0\\ 0,FP+TP=0 \end{array}\right.$$ (8)

$$PSR=\frac{TP}{TP+FN}$$ (9)

where *FP* is the number of false selected variables (false positive), *TP* represents the number of correctly selected variables (true positive), and *FN* is the number of false dropped variables.

### 3.3 Simulation results

First, we assess the selection performance for IVs. Table 1 presents the selection results of IVs in the simulation with different sample sizes and censoring rates under parameter settings in Scenario 1. As the sample size increased, the performance of all four methods became better. The LASSO and MCP approach yielded the best (and similar) PSR while the FDR of the MCP approach is lower than that of the LASSO approach as shown in Table 1. According to the results presented in Table 1, we decided to use the MCP-based logistic regression as the IV selection method.

Based on the IVs selected, we conducted the two-stage test and estimated the mediation effect. We evaluated the accuracy of the identification of mediators based on the proposed approach and made a comparison with the unadjusted approach (classical method).

Table 2 presents the FDR and PSR of mediator detection based on Sobel’s test, joint test, and the bootstrap test through the proposed approach and the classical approach (without adjustment of potential confounders) under the parameter setting in Scenario 1. As shown in Table 2, in general, compared with the classical unadjusted approach, the proposed method yielded a more reliable FDR level and higher PSR.

**TABLE 2:** FDR and PSR in the mediation test by the proposed method, PS method, CoxMKF, and classical method with unmeasured confounders.

Method		*n* = 200		*n* = 500		*n* = 800	
		FDR	PSR	FDR	PSR	FDR	PSR
Censoring rate: 20%							
Proposed method	Sobel	0.0011	0.639	0.0013	0.911	0.0014	0.999
	Joint	0.0012	0.695	0.0016	0.924	0.0016	1.000
	Boot	0.0013	0.811	0.0014	0.989	0.0018	1.000
PS-based method	Sobel	0.0014	0.501	0.0016	0.595	0.0019	0.889
	Joint	0.0014	0.589	0.0018	0.612	0.0021	0.898
	Boot	0.0019	0.601	0.0022	0.756	0.0021	1.000
Classical	Sobel	0.0016	0.361	0.0019	0.601	0.0021	0.880
	Joint	0.0015	0.345	0.0023	0.615	0.0025	0.910
	Boot	0.0015	0.685	0.0023	0.784	0.0027	1.000
CoxMKF	—	0.0013	0.675	0.0017	0.794	0.0019	0.981
Censoring rate: 40%							
Proposed method	Sobel	0.0012	0.690	0.0015	0.995	0.0019	1.000
	Joint	0.0013	0.705	0.0016	0.999	0.0021	1.000
	Boot	0.0014	0.85	0.0020	1.000	0.0023	1.000
PS-based method	Sobel	0.0012	0.475	0.0019	0.615	0.0025	0.901
	Joint	0.0015	0.490	0.0019	0.652	0.0028	0.989
	Boot	0.0019	0.555	0.0025	0.851	0.0028	0.999
Classical	Sobel	0.0009	0.355	0.0018	0.621	0.0022	0.911
	Joint	0.0014	0.385	0.0019	0.672	0.0025	0.925
	Boot	0.0018	0.695	0.0024	0.885	0.0026	0.981
CoxMKF	—	0.0015	0.655	0.0022	0.875	0.0026	0.996
Censoring rate: 60%							
Proposed method	Sobel	0.0015	0.623	0.0015	0.872	0.0016	0.999
	Joint	0.0017	0.635	0.0019	0.845	0.0022	1.000
	Boot	0.0021	0.758	0.0022	0.929	0.0024	1.000
PS-based method	Sobel	0.0013	0.442	0.0022	0.569	0.0025	0.885
	Joint	0.0017	0.453	0.0019	0.570	0.0025	0.930
	Boot	0.0019	0.552	0.0022	0.652	0.0026	0.965
Classical	Sobel	0.0014	0.345	0.0021	0.571	0.0025	0.884
	Joint	0.0015	0.430	0.0022	0.565	0.0028	0.925
	Boot	0.0019	0.595	0.0022	0.796	0.0026	0.960
CoxMKF	—	0.0017	0.550	0.0022	0.815	0.0028	0.966

As presented in Table 2, under a fixed censoring rate, the proposed method with the bootstrap test yielded the best performance. However, with the increase in the sample size, the performance of the proposed method with Sobel’s, joint, and bootstrap approaches tended to be close to each other. With the increase in the censoring rate, the FDR and PSR levels of the proposed method and classical method with all three hypothesis test approaches became worse. With unmeasured confounders, the performance of the PS method and the classical method is similar, and both were worse than the proposed method.

The performance of the proposed method, PS method, and unadjusted method in estimation of indirect effects with unknown confounders under parameter setting in Scenario 2 is presented in Table 3. In general, with the increase in sample size, the MSE of all approaches decreased. The MSE became larger when the censoring rate increased in both scenarios. The estimation of the mediation effect obtained with the proposed method was close to the set level and got closer when the sample size became larger. While the estimation obtained with the unadjusted classical approach and the PS approach was quite biased, as shown in Table 3.

**TABLE 3:** Estimation of the mediation effect with 1,000 potential covariates (including the confounders, IVs, and exposures) and *γ*=(1.2,0.8,1.5,0,0,…,0), *λ*=(0.8,1.2,0,1.5,0,…,0) with unmeasured confounders.

Cens. rate (%)	(γ, λ)	n = 200					n = 500				n = 800				
		IV ^a^	MKF ^b^	PS ^c^	Class. ^d^	IV ^a^		MKF ^b^	PS ^c^	Class. ^d^		IV ^a^	MKF ^b^	PS ^c^	Class. ^d^
20	(1.2,0.8) = 0.96 (MSE)	0.956 (0.0212)	1.786 (0.2125)	1.756 (0.2875)	2.446 (0.4727)	0.964 (0.0135)		1.806 (0.1890)	2.039 (0.1721)	2.260 (0.3843)		0.974 (0.0062)	1.428 (0.1303)	2.075 (0.1477)	2.046 (0.2089)
	(0.8,1.2) = 0.96 (MSE)	0.974 (0.026)	1.755 (0.2248)	1.985 (0.4813)	2.215 (0.3927)	0.967 (0.0170)		1.843 (0.1552)	2.075 (0.3239)	2.082 (0.3126)		0.952 (0.0066)	2.363 (0.1242)	1.866 (0.2621)	1.943 (0.2220)
	(1.5,0) = 0 (MSE)	—	—	0.4987 (0.3981)	0.963 (0.3125)	0.007 (0.0079)		0.806 (0.2011)	0.767 (0.3108)	0.556 (0.3128)		—	1.045 (0.1731)	1.062 (0.1541)	0.765 (0.2266)
	(0,1.5) = 0 (MSE)	—	—	—	0.742 (0.3685)	—		—	0.752 (0.3202)	0.756 (0.2956)		—	—	0.770 (0.2544)	0.805 (0.2515)
	(0,0) = 0 (MSE)	—	0.8861 (0.4650)	0.8958 (0.4685)	—	—		—	—	0.877 (0.3013)		—	—	—	0.687 (0.2448)
40	(1.2,0.8) = 0.96 (MSE)	0.947 (0.0210)	1.915 (0.4464)	1.896 (0.4206)	1.870 (0.4780)	0.961 (0.0125)		1.737 (0.2376)	1.770 (0.2735)	1.964 (0.3765)		0.961 (0.0071)	1.843 (0.1579)	1.786 (0.2177)	2.045 (0.2934)
	(0.8,1.2) = 0.96 (MSE)	0.979 (0.0244)	1.752 (0.4711)	1.940 (0.4112)	1.829 (0.4893)	0.968 (0.0122)		1.944 (0.2633)	1.638 (0.3715)	2.260 (0.3977)		0.958 (0.0063)	1.928 (0.1866)	1.944 (0.2379)	2.199 (0.3045)
	(1.5,0) = 0 (MSE)	—	—	0.915 (0.4089)	0.892 (0.5181)	—		1.802 (0.2150)	0.956 (0.3480)	0.737 (0.3519)		—	—	0.731 (0.1852)	0.888 (0.2575)
	(0,1.5) = 0 (MSE)	—	—	—	0.745 (0.4714)	—		—	—	0.944 (0.3850)			0.687 (0.1990)	0.553 (0.3541)	0.632 (0.2843)
	(0,0) = 0 (MSE)	—	0.8529 (0.5631)	0.745 (0.4556)	0.843 (0.4884)	—		—	0.906 (0.3515)	—		—	—	—	—
60	(1.2,0.8) = 0.96 (MSE)	0.967 (0.0184)	1.962 (0.3517)	1.785 (0.5311)	1.752 (0.4939)	0.985 (0.0124)		1.677 (0.2820)	2.446 (0.3126)	2.075 (0.3587)		0.974 (0.0091)	1.027 (0.1445)	2.379 (0.2448)	2.275 (0.2301)
	(0.8,1.2) = 0.96 (MSE)	0.954 (0.0244)	2.284 (0.4477)	1.697 (0.5456)	1.715 (0.5822)	0.973 (0.0144)		2.105 (0.3349)	1.962 (0.4822)	1.942 (0.3651)		0.982 (0.0120)	1.068 (0.1677)	1.973 (0.2291)	1.774 (0.2365)
	(1.5,0) = 0 (MSE)	—	—	0.732 (0.6376)	0.786 (0.2171)	—		—	0.893 (0.4489)	0.788 (0.1781)		—	—	0.820 (0.3264)	0.687 (0.1244)
	(0,1.5) = 0 (MSE)	0.002 (0.0022)	0.175 (0.0521)	0.761 (0.4411)	0.698 (0.5248)	—		1.055 (0.2365)	—	0.797 (0.4405)		—	0.925 (0.1281)	—	0.756 (0.2934)
	(0,0) = 0 (MSE)	—	—	0.715 (0.2102)	0.441 (0.2016)	—		—	0.858 (0.3254)	0.965 (0.3182)		—	—	—	0.246 (0.2067)

^a^ The proposed IV-based method.

^b^ The CoxMKF approach.

^c^ The PS-based approach.

^d^ The classical method.

Table 4 presents the simulation results illustrating the performance of the proposed method and classical approach in estimation of indirect effects with parameter setting in Scenario 3. When the number of covariates (as well as the number of mediators) increased, the proposed method still yielded good performance. The estimation of indirect effects by classical approach is seriously biased.

**TABLE 4:** Estimation of the mediation effect with 3,000 potential covariates (including the confounders, IVs, and exposures) and *γ*=(1.2,0.8,-1.2,1.2,1.5,0,0,…,0), *λ*=(0.8,1.2,0.8,-0.8,0,1.5,0,…,0) with unmeasured confounders.

Censoring rate (%)	(*γ*, *λ*)	n = 200		n = 500		n = 800	
		Proposed	Classical	Proposed	Classical	Proposed	Classical
20	(1.2,0.8) = 0.96 (MSE)	0.988 (0.0274)	2.022 (0.4403)	0.968 (0.0144)	1.506 (0.3495)	0.945 (0.0079)	1.346 (0.2063)
	(0.8,1.2) = 0.96 (MSE)	0.986 (0.0216)	1.922 (0.5433)	0.972 (0.0193)	2.098 (0.5201)	0.952 (0.0101)	1.948 (0.4740)
	(-1.2,0.8) = -0.96 (MSE)	−0.973 (0.0291)	−1.989 (0.4406)	−0.953 (0.0242)	−1.759 (0.3371)	−0.964 (0.0210)	−1.257 (0.1250)
	(1.2,-0.8) = -0.96 (MSE)	−1.025 (0.0373)	−1.486 (0.3771)	−0.998 (0.0209)	−1.921 (0.4259)	−0.966 (0.0112)	−1.323 (0.1867)
	(1.5,0) = 0 (MSE)	0.0011 (0.0016)	0.5765 (0.2856)	—	0.5028 (0.2458)	0.0015 (0.0018)	0.4664 (0.2295)
	(0,1.5) = 0 (MSE)	—	0.5823 (0.4581)	—	0.6756 (0.2789)	—	—
	(0,0) = 0 (MSE)	0.0063 (0.0023)	0.4903 (0.2730)	0.0020 (0.0017)	—	—	0.4317 (0.2122)
40	(1.2,0.8) = 0.96 (MSE)	0.956 (0.0317)	1.698 (0.4401)	0.967 (0.0707)	2.036 (0.5011)	0.963 (0.0107)	1.460 (0.3975)
	(0.8,1.2) = 0.96 (MSE)	0.988 (0.0247)	2.759 (0.5945)	0.971 (0.0170)	1.245 (0.5302)	0.968 (0.0092)	2.041 (0.4947)
	(−1.2,0.8) = -0.96 (MSE)	−0.969 (0.0314)	−2.015 (0.4982)	−0.649 (0.0278)	−1.783 (0.5231)	−0.969 (0.0097)	−1.258 (0.3451)
	(1.2,−0.8) = -0.96 (MSE)	−0.970 (0.0393)	−1.812 (0.4823)	−0.965 (0.0289)	−1.966 (0.3833)	−0.958 (0.0123)	−2.292 (0.4274)
	(1.5,0) = 0 (MSE)	—	0.5164 (0.2958)	-	0.3363 (0.2656)	—	0.3965 (0.2064)
	(0,1.5) = 0 (MSE)	—	0.5249 (0.2589)	0.0021 (0.0035)	0.4715 (0.3156)	0.0012 (0.0021)	0.7645 (0.2214)
	(0,0) = 0 (MSE)	—	0.5331 (0.3156)	0.0015 (0.0023)	0.6612 (0.2561)	—	0.5312 (0.2164)
60	(1.2,0.8) = 0.96 (MSE)	0.966 (0.0345)	1.799 (0.5763)	0.958 (0.0753)	1.952 (0.5089)	0.964 (0.0194)	1.619 (0.4045)
	(0.8,1.2) = 0.96 (MSE)	0.941 (0.0283)	2.544 (0.4494)	0.967 (0.0214)	1.558 (0.4994)	0.952 (0.0113)	1.797 (0.4961)
	(−1.2,0.8) = -0.96 (MSE)	−0.982 (0.0312)	−1.523 (0.5011)	−0.979 (0.0258)	−1.896 (0.4492)	−0.958 (0.0198)	−1.897 (0.3789)
	(1.2,−0.8) = -0.96 (MSE)	−1.001 (0.0395)	−1.298 (0.4864)	−0.982 (0.0289)	−1.750 (0.4898)	−0.974 (0.0144)	−2.905 (0.4477)
	(1.5,0) = 0 (MSE)	0.0016 (0.0035)	—	0.0013 (0.0030)	0.4715 (0.3240)	—	—
	(0,1.5) = 0 (MSE)	—	0.7267 (0.3440)	0.0026 (0.0051)	0.6488 (0.3256)	—	0.6473 (0.3009)
	(0,0) = 0 (MSE)	—	0.5164 (0.3516)	—	0.6488 (0.2756)	—	0.5411 (0.2288)

## 4 Empirical study

In this empirical study, we aimed to identify potential DNA methylation markers that may act as a mediator between smoking and the overall survival (OS) outcome of patients with squamous cell lung cancer. Data were obtained from the project LUSC of The Cancer Genome Atlas (TCGA) database (https://portal.gdc.cancer.gov/). A total of 754 patients with squamous cell lung cancer were included in the analysis. The basic features of the included patients are presented in Table 5. In summary, a total of 305 patients died during follow-up, and the median survival time was 54.4 (44.9–61.4) months.

**TABLE 5:** Basic features of the included cases.

Feature		Vital status		*P*
		Alive (*n* = 449)	Dead (*n* = 305)	
Smoking	No	337 (75.1%)	217 (71.1%)	0.267
	Yes	112 (24.9%)	88 (28.9%)	
Stage	I-II	399(88.9%)	218(71.5%)	<0.001
	III-IV	50(11.1%)	87(28.5%)	
Radiation	No	409 (91.1%)	255 (83.6%)	0.003
	Yes	40 (8.9%)	50 (16.4%)	
Gender	Female	198 (44.1%)	120 (39.3%)	0.222
	Male	251 (55.9%)	185 (60.7%)	
Age (in years)		66.0 ± 9.4	67.4 ± 10.0	0.046

DNA methylation is an endogenous modification process present in eukaryotes that involves the transfer of a methyl group to the C5 position of cytosine to form 5-methylcytosine. A published study has indicated that DNA methylation plays an important role in tumorigenesis and can trigger the initiation of cancer by reactivating silenced oncogenes (Bolger et al., 2014). Environmental factors also have a great impact on DNA methylation levels, especially long-term smoking or exposure to second-hand smoke, which may significantly alter DNA methylation levels (Lee and Pausova, 2013).

In this empirical study, we aim to identify DNA methylation CpGs that act as mediators between smoking and OS in patients with squamous cell lung cancer. Considering that there might be confounders and may not be measured during the data collection process, we applied the proposed IV-based two-stage approach with different mediation test methods to explore potential mediators controlling for potential confounders. We used the smoking status (yes or no) as exposure and survival prognosis (live or dead, and survival time in months) as an outcome. DNA methylation signatures were regarded as potential high-dimensional mediators. The results are proposed in Table 6. As shown in Table 6, *P*_*Sobel*_ refers to the *p*-values obtained with the Sobel’s test, *P*_*Joint*_ refers to the *p*-values obtained with the joint test, and *P*_*Boot*_ refers to the *p*-values obtained with the bootstrap test, and all *p*-values were corrected with the Bonferroni approach. The hazard ratios and corresponding 95%CIs for mediators cg27042065, cg21926276, and cg26387355 were 1.097 (1.016 and 1.1841), 1.254 (1.114 and 1.411), and 1.171 (1.067 and 1.285), respectively. The selected IVs using the proposed method include cg06320150, cg16205058, cg02089348, cg07964097, and cg02599390.

**TABLE 6:** Results of the mediation effect analysis based on the proposed method with empirical data.

CpG	λ	γ	Mediation effect (95%CI)	*P*_*Sobel*_	*P*_*Joint*_	*P*_*Boot*_	Chromosome (start, end)
cg27042065	−0.050	−1.870	0.093(0.016, 0.169)	0.123	0.055	0.049	chr12 (6959656, 6959658)
cg21926276	−0.058	−3.902	0.226(0.108, 0.344)	0.001	<0.001	<0.001	chr11 (2035254, 2035256)
cg26387355	−0.057	−2.786	0.158(0.065, 0.251)	0.006	<0.001	<0.001	chr12 (131979065, 131979067)

Since, in general, smoking increases the risk of lung cancer and reduces overall survival outcome, followed by Luo et al. (2020) and Yu et al. (2021), we also only presented those CpGs with *λγ*>0. More complete results (with those mediators with *λγ<*0) are available in Supplementary Section S2.

The identified methylation signature cg27042065 is located in gene CDCA3 which is found to be associated with the survival prognosis and may act as a potential therapeutic marker in the treatment of on-small cell lung cancer (NSCLC) (Adams et al., 2017). cg21926276 is located in gene H19 which is well-known as a tumor-related gene in multi cancers including NSCLC (Wang et al., 2021). cg26387355 is located in gene LOC338797 which is also been found to be associated with lung cancer prognosis (Song and Yang, 2018). These also suggested that the DNA methylation signatures identified with the proposed approach were reliable.

We also compared the results using the CoxMKF approach (Tian et al., 2022), as presented in the Supplementary Section S2. Most of the identified CpGs with two methods were consistent.

## 5 Discussion

Epigenetic research is often conducted based on data collected in an observational study, and researchers are often interested in the role of epigenetic modifiers between exposures and health outcomes, thus mediation analysis is critical. Classical mediation analysis often assumes that there are no confounders, however, this assumption is hard to behold in the observational epigenetic study (Boyko, 2013). To address this issue, several methods have been proposed (Armstrong, 2012). Existing methodologies controlling confounders in mediation analysis usually assume that potential confounders, at least the most important ones, were known or measured. However, this assumption was also difficult to behold in practice. Therefore, in this study, we proposed a statistical tool to solve the issue of the control of unmeasured confounders.

In this study, we addressed the problem of adjusting for unmeasured confounders by applying the IV approach. The simulation study was conducted to decide the optimized variable selection method. We used three hypothesis testing methods including the Sobel’s test, joint test, and the bootstrap test to test the significance of the mediation effect. The results of the simulation study has suggested that the proposed approach can correctly estimate the indirect effects and yielded good performance in hypothesis testing considering the FDR and PSR rates. As shown in the simulation study, when unknown confounders existed, the estimation of the PS approach would be biased. Our approach does not require researchers to pre-obtain the measurements of all or most of the major potential confounders. This may especially benefit exploration studies. Our methods require less information, and also can well control the confounder, which is one of the strength of our methods compared with other existing methods. The empirical study based on the DNA methylation measurement of lung cancer patients also illustrated its application in real data analysis. Larger sample size may enhance the identification of mediators and the estimation of indirect effects. However, a higher censoring rate may introduce bias in identification of mediators and the estimation of indirect effects.

Though in the empirical study, five CpGs were selected as IVs; however, in the proposed approach, we did not limit the IV to only be genetic variations, the IVs can be either genetic variations or clinical or social-demographic features. The selection of IVs is completely driven by the data or the algorithm. Thus our approach may be classified as based on the general IV method. This is a difference between our approach and the Mendelian randomization method.

In addition, the Mendelian randomization is a special case of the IV approach. In the general sense of the IV method, any type of variable can be used as an IV, while Mendelian randomization specifically refers to the use of genetic variation as IV to infer a causal relationship between exposure factors and outcomes. In our approach, we did not limit the IV to only be genetic variations, thus our approach may be classified as based on the general IV method.

In the establishment of the proposed method, several assumptions have been mentioned in Section 2.3. Those assumptions were made following other published approaches (VanderWeele, 2011; Huang and Yang, 2017; Yu et al., 2021; Tian et al., 2022) to ensure the identification of mediating effects. In practical data analysis, researchers can check the assumptions through regression analysis. In practical data analysis, serious violation of those assumptions may lead to biased estimation and incorrect identification of mediators (VanderWeele, 2011; Huang and Yang, 2017).

In addition, the time needed for three hypothesis testing methods varies. In general, with 10,000 covariates (including three true mediators) and sample size equals to 300, the needed time for Sobel’s test was 23.73 s, for joint test 31.57 s, and for bootstrap method 33.36 s (OS: windows 10; Processor: Intel Core i7-8850H CPU @2.60 GHz; RAM: 16.0 GB). The simulation study suggested that the bootstrap method obtained the optimized FDR and PSR and was not affected much by the sample size. While the FDR and PSR for the other two methods also become better when the sample size increased. Therefore, we may suggest that when the sample size is not very large, the bootstrap method may obtain more robust results; while when the sample size is relatively large, the performance of all three methods are similar, but Sobel’s test and the joint test may need much less time.

Our method was proposed under the assumption that key confounders were unmeasured. In the additional simulation study, we also explored the statistical performance of the proposed method in the situation where all key confounders were measured. As shown in the Supplementary Table S1 in the supplementary file, the proposed method also yielded good statistical performance, so as the PS-based method and the CoxMKF method. These results and the simulation results in the main text together suggested that the proposed method can be a useful statistical tool for high-dimensional mediator analysis controlling the influence of potential confounders. Also, the advantage of the proposed method is that it can control the influence of potential confounders even when the key confounders were not measured. In addition, this may make our method a good alternative to other methods used in the analysis of high-dimensional mediated effects in survival data controlling confounding factors.

The results of the empirical study also suggested that the results obtained with the proposed method are reasonable. Though the mediators selected by the proposed method and the CoxMKF method (as shown in the Supplementary Section S2) were not completely the same; however, many of the selected mediators were consistent.

Mediation analysis provides evidence for exploring the relationship between disease and exposure by determining intermediate variables in the pathway in epigenetic studies (Vo et al., 2022). In observational epigenetic studies, confounders are inevitable and may not always be able to be measured. Ignoring the influence of confounders may easily lead to biased estimation of the effect or miss-detection of mediating factors (VanderWeele and Chiba, 2014; Valente et al., 2017; Stuart et al., 2021). Among the commonly used methods in confounder control, the IV method can better control the influence of unknown confounding factors, thus is widely used in observational data analysis.

During the last decade, there were other published methods focusing on high-dimensional mediator analysis and unmeasured confounders. Wang et al. (2017) proposed a method under the framework of linear models to solve the issue of multiple testing with unmeasured confounders (Wang et al., 2017). This method also provided useful tools for dealing with unmeasured confounders. The difference between our approach and Wang et al.’s is that their approach was focusing on continuous outcomes and has not yet expanded to survival data. It would be of potentials to expand their approaches into time-to-event outcomes. Zhang et al. (2021b) established a high-dimensional mediator identification approach for survival data based on the SIS method and a de-biased LASSO inference procedure (Zhang et al., 2021a) and this method was further extended by Perera et al. (2022) (Perera et al., 2022). Their methods can be useful in the identification of high-dimensional mediators for survival data; however, their methods also did not take the issue of unmeasured confounders into consideration. In addition, Liu et al. (2022) have proposed a novel powerful DACT approach to exploring high-dimensional mediating effects adjusting for confounders (which require all confounders were known) (Liu et al., 2022). It also would be of great values to expand the application of their method and ideas into the situation with unmeasured confounders and survival outcomes.

Still, there are several issues that are remained. First, we only considered the situation that the exposure factor has only two levels. Methodologies for ordinal, multi-levels, and continuous exposure factors are still needed to be developed. Our approach does not address the issue that confounders affect the relationship between mediators and the outcome, or exposure and the mediator. Future works focusing on these issues would also be of interest.

## 6 Conclusion

In general, the proposed method has good statistical performance and can be a useful statistical tool for high-dimensional mediation analysis in the observational study with unmeasured confounders. Our approach may promote the application of high-dimensional mediation effect analysis in observational epigenetic studies.

## Data Availability

The lung cancer data used for the empirical study can be obtained by any researcher at https://portal.gdc.cancer.gov/ without any limitations. The proposed method is implemented using the R-programming language, and the corresponding R codes can be obtained at https://github.com/LiuWeiVivian64/HDMA_IV.git.
