# Supplementary material for: Instrumental variable-based high-dimensional mediation analysis with unmeasured confounders for survival data in the observational epigenetic study
Source: Front Genet. 2023 Feb 2;14:1092489. doi: 10.3389/fgene.2023.1092489 (PMC9932046; doi:10.3389/fgene.2023.1092489)
Supplement: Supplementary file 1 [file DataSheet1.docx]

Supplementary Material

# Additional simulation

The proposed method was developed under the assumption that unmeasured confounders exist. In the main text, simulation study was conducted under the same assumption. In this section, we conducted additional simulation to compare the statistical performance of the proposed method and several published approaches under the assumption that all confounders were measured. The simulation results were presented in Table S1.

The simulation parameters was set the same as in Scenario 1 in the main manuscript but the confounders were not removed.

As shown in Table S1, when all key confounders were measured, the proposed method, PS-based method and the CoxMKF approach all yielded good performance. The FDR for classical approaches was higher among all methods while its PSR was little lower.

| Table S1. FDR and PSR in mediation test by the proposed method, PS method CoxMKF and the classical method with all confounders measured. | | | | | | | |
| --- | --- | --- | --- | --- | --- | --- | --- |
| Method |  | 200 | | 500 | | 800 | |
|  |  | FDR | PSR | FDR | PSR | FDR | PSR |
| Censoring Rate: 30% | | |  |  |  |  |  |
|  | Sobel | 0.0012 | 0.644 | 0.0013 | 0.911 | 0.0015 | 0.999 |
| IV | Joint | 0.0012 | 0.675 | 0.0014 | 0.924 | 0.0016 | 1 |
|  | Boot. | 0.0014 | 0.795 | 0.0016 | 0.989 | 0.0018 | 1 |
|  | Sobel | 0.0012 | 0.64 | 0.0014 | 0.909 | 0.0016 | 0.989 |
| PS | Joint | 0.0013 | 0.689 | 0.0015 | 0.912 | 0.0019 | 0.998 |
|  | Boot. | 0.0014 | 0.796 | 0.0018 | 0.985 | 0.0019 | 1 |
|  | Sobel | 0.0012 | 0.635 | 0.0013 | 0.601 | 0.0021 | 0.988 |
| Classical | Joint | 0.0012 | 0.665 | 0.0013 | 0.615 | 0.0025 | 0.989 |
|  | Boot. | 0.0013 | 0.785 | 0.0014 | 0.974 | 0.0027 | 0.999 |
| MKF |  | 0.0013 | 0.798 | 0.0015 | 0.984 | 0.0019 | 0.998 |
| Censoring Rate: 60% | | |  |  |  |  |  |
|  | Sobel | 0.0015 | 0.624 | 0.0017 | 0.872 | 0.0016 | 0.999 |
| IV | Joint | 0.0017 | 0.635 | 0.0019 | 0.845 | 0.0022 | 0.999 |
|  | Boot. | 0.002 | 0.758 | 0.0022 | 0.92 | 0.0024 | 1 |
|  | Sobel | 0.0013 | 0.622 | 0.0022 | 0.569 | 0.0022 | 0.985 |
| PS | Joint | 0.0017 | 0.638 | 0.0019 | 0.57 | 0.0025 | 0.993 |
|  | Boot. | 0.0019 | 0.752 | 0.0022 | 0.652 | 0.0025 | 1 |
|  | Sobel | 0.0014 | 0.345 | 0.0021 | 0.57 | 0.0025 | 0.884 |
| Classical | Joint | 0.0015 | 0.634 | 0.0022 | 0.565 | 0.0028 | 0.925 |
|  | Boot. | 0.0019 | 0.759 | 0.0022 | 0.796 | 0.0026 | 0.96 |
| MKF |  | 0.0017 | 0.55 | 0.0022 | 0.815 | 0.0028 | 0.999 |

# Full results obtained in empirical study

In the main text, followed Yu et al. (2021) and Luo et al. (2020), we only presented the results for mediators with *λγ*＞0, because in general, smoking increases the risk of lung cancer and reduces overall survival. In the supplementary file, we presented the full results obtained in the empirical study obtained by the proposed approach and the CoxMKF approaches. Since the CoxMKF approach did not provide *P*-values, Table S3 only shows the mediating effects and selected mediators.

Table S1 was the result obtained by the proposed method. The selected IVs including cg06320150, cg16205058, cg02089348, cg07964097, cg02599390. Table S2 was the result obtained by Tian et al. (2022) with the CoxMKF approach. As shown in Table S2 and Table S3, most mediators identified by the two methods were consistent.

| Table S2. Results of the mediation effect analysis based on the proposed method with empirical data | | | | | | | | |
| --- | --- | --- | --- | --- | --- | --- | --- | --- |
| CpGs | Med. Eff. (95%CI) | *λ* | *γ* | *p_*bon_boot. | *p*_bon_sobel | *p*_bon_joint | Gene | Chromosome (start, end) |
| cg15292688 | -0.129(-0.221,-0.037) | -0.051 | 2.539 | <0.001 | 0.041 | 0.008 | RP11-411B10.5 | chr18 (14149418,14149420) |
| cg27042065 | 0.093(0.016,0.169) | -0.05 | -1.87 | 0.049 | 0.123 | 0.055 | CDCA3 | chr12 (6959656,6959658) |
| cg21926276 | 0.226(0.108,0.344) | -0.058 | -3.902 | <0.001 | 0.001 | <0.001 | H19 | chr11 (2035254,2035256) |
| cg26387355 | 0.158(0.065,0.251) | -0.057 | -2.786 | <0.001 | 0.006 | <0.001 | SOX1 | chr12 (131979065,131979067) |
| cg24200525 | -0.101(-0.181,-0.021) | -0.024 | 4.177 | <0.001 | 0.092 | 0.037 | SBF1 | chr22 (50897910,50897912) |
| cg07690349 | -0.132(-0.218,-0.046) | -0.075 | 1.755 | <0.001 | 0.019 | 0.002 | MUC5B | chr11 (1257658,1257660) |
| cg19587838 | -0.092(-0.171,-0.013) | -0.069 | 1.333 | <0.001 | 0.156 | 0.080 | . | chr13 (112679797,112679799) |

| Table S3. Results of the mediation effect analysis based on the CoxMKF with empirical data (Tian, et al., 2022) | | | | | |
| --- | --- | --- | --- | --- | --- |
| CpGs | Med. Eff. | λ | γ | Gene | Chromosome (start, end) |
| cg21926276 | 0.148 | -0.058 | -2.554 | H19 | chr11 (2035254,2035256) |
| cg24129177 | 0.069 | -0.056 | -1.225 | . | chr12 (131591849,131591898) |
| cg24200525 | -0.123 | -0.024 | 5.136 | SBF1 | chr22 (50897910,50897912) |
| cg07690349 | -0.134 | -0.075 | 1.789 | MUC5B | chr11 (1257658,1257660) |
